# INVESTIGATION OF TRANSCRIPTIONAL CHANGES IN IMMUNE CELL POPULATIONS OF MICE TREATED WITH RAPAMYCIN

**DOI:** 10.1093/geroni/igae098.1289

**Published:** 2024-12-31

**Authors:** Matt Yousefzadeh

**Affiliations:** Columbia University Medical Center, New York, New York, United States

## Abstract

Aging is the greatest risk factor for many chronic degenerative diseases, for example osteoarthritis, dementia and heart disease. This robs individuals of quality of life and is very costly to treat. This has led to great interest in finding rational strategies for extending health span, the years of disease-free life at the end of one’s lifespan. Theoretically, if we could treat aging itself, all of the diseases associated with old age would be prevented, delayed or attenuated simultaneously. Thus, discovering fundamental mechanisms of aging is a priority. Aging is generally thought to be driven by an accumulation of damage in cells over time. The progressive decline of immune function with age, known as immunosenescence, plays a pivot role in not only the aging process but also susceptibility to infections and reduced vaccine responses. Thus it is critical to find interventions to potentially correct the age-related deficits in immune function. The potent mTOR inhibitor rapamycin has been shown to extend lifespan in mice and reduce age-related pathologies. Over the past decade the use of rapamycin or analogs of rapamycin have been investigated for its ability to rejuvenate aged immune systems. Treatment of elderly human participants with a rapalog was able to significantly enhance antibody titer levels to the annual influenza vaccination as well as reduce immune exhaustion markers. I was able to recapitulate some of these findings in a mouse model of immune aging. However, which immune cell populations are benefited from rapamycin treatment remains to be seen. My working hypothesis is that rapamycin is able to restore function to aged T cells and possibly other immune cell types. In order to test this hypothesis 18-month-old WT C57BL/6 mice were placed onto chow containing encapsulated rapamycin (126 ppm) or control chow for 7 months. Spleens were harvested from these mice and 5-month-old young control mice (n=3-4 per sex per group). Splenic samples are currently undergoing single-nuclei RNA sequencing to investigate the effect of rapamycin on gene expression changes in different immune cell populations of the spleen and address the following questions: Question 1: What are the differences in gene expression profiles in the different immune cell populations between young versus old splenic samples? Question 2: Does treatment with rapamycin in old mice restore a more “youthful” expression profile to all immune cell types or just a subset of them? Question 3: Are there sex-based differences in the gene expression patterns of aged immune cells or in response to rapamycin treatment?

